# Beyond Cognition: Cognitive Re-Education’s Impact on Quality of Life and Psychological Well-Being in People with Multiple Sclerosis—A Narrative Review

**DOI:** 10.3390/neurosci6030064

**Published:** 2025-07-15

**Authors:** Nicola Manocchio, Chiara Moriano, Anna D’Amato, Michela Bossa, Calogero Foti, Ugo Nocentini

**Affiliations:** 1 Physical and Rehabilitation Medicine, Clinical Sciences and Translational Medicine Department, Tor Vergata University, 00133 Rome, Italy; morianochiara@gmail.com (C.M.); damatoanna.92@gmail.com (A.D.); bossa.michela@gmail.com (M.B.); foti@med.uniroma2.it (C.F.); 2 PhD Course in Tissue Engineering and Remodeling Biotechnologies for Body Function, Tor Vergata University, 00133 Rome, Italy; 3 Behavioural Neuropsychology, IRCCS “Santa Lucia” Foundation, 00179 Rome, Italy

**Keywords:** Multiple Sclerosis, cognitive impairment, rehabilitation, Quality of Life, narrative review, psychological well-being, physical and rehabilitation medicine

## Abstract

Cognitive impairment is a prevalent and disabling feature of multiple sclerosis (MS), significantly impacting patients’ quality of life (QoL) and psychological well-being. Despite its clinical relevance, there are currently no approved pharmacological treatments for cognitive deficits in MS, highlighting the need for effective non-pharmacological interventions. This narrative review explores evidence from studies evaluating the efficacy of cognitive re-education (CR) approaches (including traditional, group-based, computer-assisted, virtual reality, and innovative methods such as music therapy) on cognitive and QoL outcomes in people with MS. The findings demonstrate that while CR consistently influences cognitive domains such as memory, attention, and executive function, its effects on QoL are more variable and often depend on intervention type, duration, and individual patient characteristics. Notably, integrative approaches like virtual reality and music therapy show promising results in enhancing both cognitive performance and psychosocial well-being. Several studies report that cognitive gains are accompanied by improvements in mental health and functional QoL, particularly when interventions are tailored to individual needs and delivered within multidisciplinary frameworks. However, some interventions yield only limited or transient QoL benefits, underlining the importance of personalized, goal-oriented strategies that address both cognitive and psychosocial dimensions. Further research is needed to optimize intervention strategies and clarify the mechanisms linking cognitive and QoL outcomes.

## 1. Introduction

Multiple sclerosis (MS) is a chronic, autoimmune, inflammatory and degenerative disease of the central nervous system (CNS). MS causes a loss of myelin in the white matter of the cerebral hemispheres, brain stem, cerebellum, and spinal cord and the degeneration of axons and neuronal loss in all CNS areas and structures [1]. People with MS (pwMS) can suffer from motor, sensory, cognitive, and behavioral disabilities [2,3].

Cognitive impairment (CI) is a common feature of MS, affecting approximately 43–70% of patients across all disease stages [4]. The most frequently observed cognitive deficits include slowed information processing speed, inefficient learning, and long-term memory impairments—encompassing difficulties in acquiring new information and recalling it later. Additionally, impairments in attention, working memory, executive functions, and visuospatial abilities are also prevalent [5].

CI significantly impacts Quality of Life (QoL) in pwMS, at a familial, social, and professional level [6]. The early onset of MS and its persistence throughout life further amplify the disabling effects of CI and its impact on QoL [7]. Research has shown that pwMS with CI encounter greater difficulties in fulfilling occupational demands, resulting in higher unemployment rates compared to cognitively intact individuals or healthy controls [8]. This occupational disability is closely linked to CI, which represents a strong predictor of high rates of joblessness in this population [9]. Furthermore, CI negatively affects social engagement, leading to decreased participation in social activities [10]. Individuals with MS and CI often struggle with the emotional and social nuances required for effective interpersonal interactions, as deficits in social cognition (the ability to understand and respond to social cues) are prevalent in pwMS. This impairment can exacerbate feelings of isolation and impact existing relationships, ultimately leading to a decline in QoL [11]. Moreover, an increased prevalence of psychological burden often accompanies CI. While CI primarily refers to deficits in domains such as information processing speed, memory, attention, executive functions, and visuospatial abilities, psychological burden encompasses mood disturbances (e.g., depression, anxiety), emotional distress, and related psychosocial challenges [12]. In some cases, CI appears early in the disease’s course, sometimes even before overt mood disturbances, suggesting it may precede psychological symptoms. Conversely, mood disorders such as depression and anxiety can exacerbate cognitive symptoms, particularly in attention and memory, making it challenging to disentangle cause and effect [13].

The cognitive declines experienced by pwMS may not be solely related to overt neurological damage but can also stem from a complex interplay of psychosocial factors impacting their coping mechanisms and overall mental health [14]. As such, the implications of CI extend beyond mere cognitive decline; they reverberate throughout all life domains, necessitating a comprehensive approach for pwMS [15].

Despite the significant impact of CI on daily functioning, QoL, and overall well-being, there are currently no approved pharmacological treatments for it [16,17]. Cognitive re-education (CR) emerged as a novel therapeutic intervention aimed at improving or restoring cognitive functions compromised by brain injuries, neurodegenerative diseases, or psychiatric disorders [18,19]. The CR goal is not only to recover lost functions but also to develop alternative strategies to manage and adapt to cognitive deficits. Treatment is based on targeted exercises, mental stimulation techniques, emotional, and educational support and may also include the use of advanced technologies to facilitate learning and adaptation [20,21]. CR is particularly effective when initiated early and can significantly improve patients’ QoL. Furthermore, CR not only targets patients but also their families, providing them with tools to better understand their loved one’s condition and support the recovery process [22,23].

This narrative review aims to outline the evidence supporting CR in MS, focusing on its impact on both cognitive performance and, importantly, QoL outcomes, while also exploring the relationship between cognitive and psychological changes. This assessment will encompass a wide range of interventions, including traditional CR, group-based CR, computer-based CR, and innovative approaches such as music therapy. Subsequently, the possible correlation between the addressed outcomes will be investigated. Thus, we aim to provide an overview of the effects of various CR approaches and highlight the potential of CR to improve the lives of pwMS.

## 2. Traditional CR

Traditional CR emphasizes compensatory strategies to help individuals adapt to CI rather than restoring lost abilities. Techniques include internal aids like mnemonics, mental reviewing, and error-free learning, as well as external aids such as diaries, calendars, and electronic devices. These tools aim to enhance memory retention, attention, and concentration while providing practical solutions for daily cognitive challenges. Additional methods like repetition-based learning and structured exercises further support memory encoding and recall [5]. CR can be delivered either via groups or 1-to-1 sessions. Individual CR offers personalized and flexible interventions tailored to specific cognitive, emotional, and behavioral difficulties, fostering a close therapist–patient relationship. However, it may lack social stimulation compared to group CR, which provides interaction and mutual support among participants [24,25].

Lincoln et al. [26] divided patients into three groups: a control group that did not receive any intervention, an assessment group that underwent a detailed cognitive evaluation, the result of which was fed back to the healthcare staff involved in their care, and a treatment group that received both the cognitive assessment (as well as the assessment group) and a targeted re-educational program aimed at mitigating the impact of identified cognitive impairments. Cognitive assessment was focused on measures of memory, attention, and executive functioning (using the Wechsler memory scale revised, the Stroop neuropsychological screening test, and the Modified Card Sorting Test), also using an everyday memory questionnaire (EMQ—revised version). Further assessments were selected based on patients’ performance and included the Test of Everyday Attention (TEA), Behavioral Assessment of the Dysexecutive Syndrome (BADS), the Doors and People Test, the Recognition Memory Test (RMT), and the Verbal and Spatial Reasoning Task (VESPAR). The intervention included training in the use of external memory aids such as diaries, calendars, notebooks, and lists, as well as specific techniques to support memory functioning, in order to reduce the impact of cognitive impairments in the activities of daily living. Despite these efforts, no significant improvements were observed in cognitive symptoms [measured by the EMQ], mood [assessed via the General Health Questionnaire (GHQ-28)], or QoL (evaluated using the SF-36) across any of the groups at 4-and 8-month follow-ups.

A comparable approach, though with distinct primary objectives, was employed in the study by Brenk et al. [27]. This investigation involved recruiting both pwMS and healthy controls to participate in a non-specific, six-week home-based cognitive training program. Each week, participants received a set of modules containing exercises for memory and attention, derived from the book “Gripsgymnastik/Brain-Gym.” The attention tasks targeted skills such as differentiation, comparison-making, and mental counting, while memory tasks focused on encoding and recalling images, figures, word lists, memories or biographical events. Additionally, visual–spatial tasks included shape and figure recognition, crosswords, and word definitions. The intervention resulted in significant improvements for pwMS in short-term and working memory, complex attention performance, and visuo-constructive abilities. Furthermore, enhancements were observed in quality of life (measured by Hamburg Quality of Life Questionnaire Multiple Sclerosis-HAQUAMS) and depression was assessed via Beck’s Depression Inventory (BDI). However, a notable decline in long-term verbal memory was reported following the six-week training period. Lastly, Goverover et al. [28] investigated the self-generation learning program (self-GEN trial), a behavioral intervention designed to enhance learning and memory through self-generation techniques and metacognitive strategies. The intervention builds on evidence that self-generated information is better retained than externally provided information (e.g., items read or heard). Participants in the self-GEN treatment group and the placebo control group underwent six individualized 60 min sessions. During these sessions, participants were presented with items to learn under two conditions: provided (e.g., underlined words) and generated (e.g., fill-in-the-blank tasks). They were then asked to verbally recall the items immediately after learning them. Each session followed a consistent format but introduced new stimuli, such as creating an email account, learning a new language, or storing information on a cloud platform. While the control group participated in memory and attention training without self-generation or transfer instructions, the treatment group received targeted self-generation training. The findings revealed significant improvements in memory, attention, and functional status in the treatment group compared to the placebo control group. Additionally, participants in the treatment group reported enhanced QoL and reduced depressive symptoms.

A summary of the most important traditional CR features is provided in Table 1. A more in-depth report of the data from all the papers analyzed for this review is available in the Supplementary Materials (Table S1).

## 3. Group CR

Group cognitive re-education (GCR) aims to enhance cognitive functioning in structured group settings [5]. A key advantage is social interaction, which fosters communication and relational skills, mutual support, and shared coping strategies. This environment can improve cognitive outcomes, emotional well-being, and QoL by reducing isolation and promoting empowerment. The choice between individual and group CR depends on patient needs, therapeutic goals, and available resources [29]. A summary of the most important group CR features is provided in Table 2, with more specifications in Table S1.

To explore this topic, Lincoln et al. [30,31] designed an intervention, the so-called CRAMMS RCT. Participants of intervention group were divided into groups of four to six people. Group sessions were held weekly for 10 weeks; interventions included restitution strategies to retrain attention and memory functions and methods to improve encoding and retrieval. In addition, participants were taught compensation strategies, such as internal mnemonics (e.g., chunking, first letter cues, and rhymes), the use of external memory aids (e.g., diaries, notebooks, and mobile phones), and methods of coping with attention and memory problems in daily life. The control group received only standard care, which involved general advice from MS nurse specialists and occupational therapists on managing cognitive difficulties. Authors reported no benefit to the QoL of the intervention group, although memory impairments slightly improved at both 6 and 12 months.

Brissart et al. [32] conducted a multicenter randomized trial to evaluate the effectiveness of the ProCog-SEP cognitive program in improving verbal and working memory in pwMS. Participants were divided into two groups. The intervention group underwent the ProCog-SEP program, which consisted of 13 group sessions, each lasting two hours, over a six-month period. The program included psychoeducational advice and cognitive exercises targeting verbal and non-verbal episodic memory, working and short-term memory, executive functions, and language. In contrast, the control group participated in non-cognitive exercises at the same frequency but did not receive cognitive strategies or advice. Results showed significant improvements in verbal and working memory in the intervention group compared to the control group, which received a neutral intervention. However, no notable improvements in QoL were observed.

In Hanssen et al.’s study [33], patients participated in cognitive group sessions designed to enhance their awareness of cognitive decline, its associated challenges, and strategies for mitigation. These sessions, structured around three two-hour meetings, focused on setting individualized objectives using the Goal Attainment Scale (GAS), identifying strategies to achieve these goals, and providing training on coping mechanisms and effective communication about multiple sclerosis. Following discharge, the control group underwent standard training, while the intervention group received additional support through six brief (approximately 10 min) telephone sessions. These calls were aimed at monitoring progress toward the established objectives. At the conclusion of the intervention, both groups demonstrated improvements from baseline to 4 and 7 months on the Global Executive Composite Score and the Metacognitive Index of the BRIEF-A. However, only the intervention group exhibited significant enhancements in psychological well-being, as well as in anxiety and depression symptoms. Furthermore, the intervention group showed notable improvements in health-related QoL, highlighting the added value of targeted follow-up interventions alongside standard re-education.

Jogen et al. conducted a study to assess the efficacy of a 3-day intensive sociocognitive intervention known as Can Do Treatment (CDT) [34]. This multidisciplinary program, grounded in social cognitive theory, aims to identify and address the so-called “stressors” that restrict pwMS in their physical, psychological, or social roles related to their disease and associated limitations. The intervention included large group sessions, small group discussions, individual counseling, an evening at the theater, and an optional joint activity at the start of each day. The control group did not receive the CDT intervention but instead underwent cognitive testing and was offered the opportunity to participate in CDT at a later time. Results indicated an improvement in the Multiple Sclerosis Self-Efficacy Scale-Control (MSSES-C) scores at one month after treatment for patients who participated in CDT, with this trend persisting at six months, although the changes were not statistically significant. Interestingly, a gradual improvement in MSSES-C scores was also observed over time in the control group. However, no significant differences were found between the groups regarding other outcomes, including the Impact on Participation and Autonomy Questionnaire, the Multiple Sclerosis Quality of Life-54 (MSQoL-54) instrument, or the Hospital Anxiety and Depression Scale (HADS).

An innovative approach was implemented in the study by Nauta et al., where patients were randomized into three separate groups [35]. The control group received a psychoeducational appointment with a nurse specialized in MS. The second group participated in mindfulness-based cognitive therapy (four to seven participants per group), which focused on training patients to self-regulate attention and cultivate non-judgmental awareness of their moment-to-moment experiences, including emotions, thoughts, and behaviors. The third group underwent GCR once a week for nine weeks, with each session lasting 2.5 h (three to six participants per group). GCR emphasized learning and applying compensatory strategies to address issues related to processing speed, memory, executive function, and mental fatigue. Additionally, GCR targeted emotional and behavioral changes as well as grief resolution. Post-treatment outcomes revealed that both intervention groups significantly improved depression and QoL compared to controls. However, neither intervention had an effect on anxiety levels. Both treatments also enhanced mindfulness scores. Furthermore, mindfulness demonstrated improvements in executive functions and information processing speed, while GCR led to improvements in self-reported general cognitive impairment and cognitive functioning.

## 4. Computer-Based CR

Computer-based cognitive re-education (CCR) has gained significant attention in recent years due to advancements in technology and the increasing accessibility of digital tools. The widespread adoption of cutting-edge computer systems and IT devices has made these technologies more familiar to the general population, facilitating their integration into various applications, including healthcare and CR [5]. Numerous studies have investigated the potential of these approaches, demonstrating how they can be effectively implemented in CR programs for pwMS. A summary of the most important group CR features are reported in Table 3, with more specification in Table S1.

Stuifbergen et al. conducted a multicenter study to evaluate the effectiveness of the CCR program MAPSS_MS [36]. The intervention group engaged in cognitive exercises and games using the Lumosity platform, accessible via standard web browsers, targeting common cognitive deficits in pwMS, such as memory, attention, flexibility, and problem-solving. The control group used “MyBrainGames”, a free program designed to improve processing speed, attention, and working memory. Both groups showed improvements over time; however, the intervention group consistently outperformed the control group. Immediately post intervention, significant gains were observed in delayed recall, sustained attention, and reduced depressive symptoms. At 3 months, the intervention group demonstrated superior cognitive performance and reported fewer depressive symptoms, maintained at 6 months. Authors also report significant improvements in QoL within the intervention group, as assessed by the Everyday Problems Test—Revised (EPT-R). These improvements are interpreted as functional enhancements in daily life, resulting from cognitive gains achieved through the intervention.

Rosti-Otajärvi et al. carried out a trial on CCR attention and working memory retraining as part of outpatient neuropsychological care [37]. The intervention consisted of 13 weekly 60 min sessions focused on targeted cognitive exercises. At six months and at one year post intervention, participants reported reduced depressive symptoms, fatigue, and psychological burden compared to baseline. Notably, the positive effects were more pronounced in patients with severe attention deficits, indicating a correlation between intervention efficacy and baseline impairment severity. However, no significant improvements in QoL were observed.

Solari et al. investigated the use of RehaCom software for CCR in pwMS [38]. This program offers personalized training for various cognitive skills, delivered in 45 min sessions twice weekly over 8 weeks. Participants were divided into an intervention group, which focused on memory and attention training, and a control group, which trained visuo-constructive and visuo-motor coordination. While both groups showed a 20% improvement in the Brief Repeatable Battery of Neuropsychological Tests (BRBNT), no significant differences were observed between participants and controls in memory or attention outcomes. However, the intervention group demonstrated improved QoL, as measured by the MSQoL-54, suggesting potential psychosocial benefits of CCR.

Hildebrandt et al. utilized a computer-based approach in which participants in the intervention group trained with a CD containing memory and working memory exercises for six weeks, practicing at least five days per week for 30 min daily [39]. The tasks, focusing on word memorization and calculations, adjusted their difficulty based on individual progress. The control group received no intervention. Post-treatment neuropsychological assessments revealed improvements in memory and working memory among participants who completed the training. However, the intervention did not affect cognitive processing speed, depression, fatigue, or health-related QoL. While the training enhanced specific cognitive functions and mitigated decline observed in the control group, its broader psychosocial impact seemed limited.

Grasso et al. utilized the Attention Processing Training Program, a CCR intervention commonly employed to address attention deficits in individuals with brain injuries [40]. The program focused on attention and memory training through tasks such as listening to descending number sequences, alphabetizing words in spoken sentences, detecting targets amidst distracting noise, and performing complex semantic categorization requiring set-switching. The control group engaged in nonspecific computer-based activities, including reading comprehension, describing images, and analyzing texts. Both interventions began one week after baseline assessment and were delivered three times weekly for three months by a trained cognitive re-educational specialist. Results indicated improvements in executive control, QoL, and depressive symptoms in participants undergoing multidisciplinary CCR.

De Giglio et al. investigated the effects of CCR using the video game *Dr. Kawashima’s Brain Training* in patients with relapsing–remitting MS [41]. The game includes exercises targeting memory, attention, calculation, and visuospatial processing. Participants played for 30 min daily, five days a week, over eight weeks. The intervention group showed significant improvements in memory and attention compared to controls. Additionally, improvements in QoL, assessed via the MSQoL-54, reinforced the potential of this CCR approach to enhance overall well-being in pwMS. However, no correlation was found between cognitive gains and QoL improvements, likely due to the small sample size and short follow-up period.

## 5. Virtual Reality

Virtual reality (VR) represents a significant technological advancement in rehabilitation, offering innovative approaches to enhance cognitive and motor functions in pwMS by creating immersive 2D/3D environments that simulate real-world settings, boosting patient engagement and motivation [42]. Research demonstrates VR’s potential to stimulate brain plasticity, improve neuropsychological deficits, and enhance motor function through enriched sensory feedback [43]. A summary of the most important VR features is provided in Table 4, with more specifications in Table S1. Leonardi et al. evaluated the Virtual Reality Rehabilitation System (VRRS) for training attention, memory, executive functions, and visuospatial skills in pwMS [44]. Participants were randomized into an intervention group, receiving VR-based training, and a control group, undergoing traditional CR. Both groups participated in three sessions per week over eight weeks. The VR group showed significant improvements in global cognitive functioning, learning ability, verbal short-term memory, lexical access, and mental health-related QoL. Mood improvements were observed in both groups.

A similar technology has also been used by Maggio et al. [45]. Authors investigated the use of BTS-NIRVANA, a semi-immersive therapeutic system for cognitive and motor re-education in patients with relapsing–remitting MS. The NIRVANA software creates a virtual environment that provides real-time feedback during multisensory exercises, which can be dynamically adjusted to individual capabilities. Participants underwent 24 CR sessions over 8 weeks, with the control group receiving traditional face-to-face therapy and the experimental group using BTS-NIRVANA. Both groups showed significant improvements in cognitive domains, motor function, QoL, and mood. However, the experimental group demonstrated greater enhancements in visual perception, spatial skills, visual short-term memory, working memory, executive functions, information processing speed, sustained attention, and functional mobility. Additionally, BDI scores revealed lower depression levels in the experimental group compared to controls.

However, while the results from VR interventions are promising, their effectiveness relative to traditional CR should be considered cautiously, considering limitations such as small sample sizes and outcome heterogeneity.

## 6. Innovative Approaches

Innovative approaches to CR have explored unconventional strategies, such as music therapy, to address cognitive impairments in pwMS. Music therapy integrates neurological and psychosocial techniques to enhance cognitive and emotional functioning. Music therapy’s efficacy is fundamentally grounded in its ability to induce neuroplastic changes through multimodal brain activation. The brain’s response to music engages multiple neural networks simultaneously, including auditory processing centers, motor regions, limbic structures, and prefrontal areas involved in executive function [46,47]. Moreover, music’s capacity to evoke powerful emotional responses stems from its direct connection to the brain’s reward system, triggering dopamine release and activating the amygdala and hippocampus. This emotional engagement seems involved in the formation of associative memory networks, where musical stimuli serve as retrieval cues that can access autobiographical memories and emotional states [48].

Impellizzeri et al. investigated the effects of music therapy on cognitive impairments in pwMS [49]. Participants were divided into a control group, receiving standard CR targeting memory, social skills, mood, and emotional awareness, and an intervention group, which combined conventional CR with neurologic music therapy (NMT). Two NMT techniques were applied: Associative Mood and Memory Training (AMMT), which uses music to evoke moods linked to autobiographical memories, facilitating recall, and Music in Psychosocial Education and Counseling (MPC), aimed at enhancing emotional expression, mood regulation, social competence, and self-awareness through guided music activities. The intervention group showed improvements in cognitive performance, QoL, and mood.

## 7. Cognitive and QoL Outcomes Relation in People with Multiple Sclerosis

The relationship between CR and QoL outcomes in pwMS reveals a complex and multifaceted interaction. Several studies have examined the impact of cognitive interventions on QoL domains, yielding mixed results (Table 5).

Several studies reported positive associations between cognitive improvements and QoL outcomes. For instance, Leonardi et al. observed that enhancements in cognitive domains like memory and learning correlated with better mental health-related QoL in the VRRS group, while Impellizzeri et al. noted that improved cognitive performance was associated with better mental QoL outcomes in the experimental group [44,49]. Grasso et al. found that improvement in executive function correlated with better general health outcomes (SF-36 General Health domain), with stronger QoL gains observed in the intervention group [40]. Stuifbergen et al. observed that improvements in cognitive measures were associated with better self-reported cognitive abilities on the PROMIS Cognitive Abilities scale, indicating a positive relation between cognition and QoL [36]. Other authors highlighted the short-term benefits of CR on QoL. Lincoln et al. reported improvements in memory problems and mood at six months, though these effects were not sustained at 12 months [30,31]. Similarly, Jongen et al. observed transient improvements in mental and physical QoL domains at one month following CDT but found no sustained differences at six months [34]. Lincoln et al. also noted that short-term QoL improvements were associated with reduced subjective cognitive complaints and improved mood, though no long-term effects were evident [26].

Other studies emphasized the variability in outcomes depending on the intervention type or individual factors. Hanssen et al. reported improvements in psychological well-being and health-related QoL exclusively within the intervention group, suggesting a direct influence of CR on QoL outcomes [33]. Brenk et al. identified associations between cognitive improvements and better QoL scores in fatigue/thinking and mood domains, while Goverover et al. linked enhanced cognition to improved self-reported QoL and functional status [27,28]. In contrast, Nauta et al. demonstrated that improvements in psychological symptoms and mindfulness skills mediated reductions in self-reported cognitive complaints but did not significantly affect objective cognitive performance, such as information processing speed. Mental QoL improvements were primarily linked to reduced psychological symptoms [35]. Similarly, in another study Lincoln et al. (2002) found no significant relationship between cognitive and QoL outcomes, while De Giglio et al. suggested that QoL improvements were more likely attributable to reduced stress from home-based training rather than direct cognitive changes [26,41].

However, some studies failed to establish a significant relationship between cognition and QoL measures. Brissart et al. found no association between improvements in verbal and working memory and changes in QoL outcomes; Hildebrandt et al. similarly reported no significant relationship between cognitive and QoL outcomes overall [32,39]. Rosti-Otajärvi et al. noted significant improvements in perceived cognitive impairments but found no correlation with group-level QoL measures; however, individual goal achievements may have contributed to subjective QoL gains [37].

Finally, certain studies highlighted weak or indirect correlations between cognition and QoL outcomes. Solari et al., for example, observed weak associations where MSQOL-54 mental health scores improved at eight weeks for the study group without corresponding gains in cognitive function scores [38]. Maggio et al., on the other hand, reported a positive correlation between improved cognitive outcomes and enhanced QoL scores following VR-based training [45].

Overall, these findings underscore the nuanced relationship between cognition and QoL in pwMS, with evidence suggesting that while some interventions yield measurable benefits for both domains, others may have limited or transient effects depending on intervention type, duration, or individual variability. CR interventions, which aim to improve or adapt to CI, exhibit varying degrees of efficacy in enhancing both cognitive performance and QoL. The outcomes of these interventions are influenced by factors such as the type of CR approach, intervention duration, and individual variability among participants. Notably, sustained benefits often require personalized, multidisciplinary, and long-term strategies that address both cognitive deficits and psychosocial needs.

## 8. Cognitive and Psychological Outcomes Relation in People with Multiple Sclerosis

The interplay between CR and psychological outcomes in pwMS has been explored in various studies, revealing both direct and indirect relationships. Several investigations highlight significant improvements in psychological well-being following cognitive interventions, often mediated by changes in cognitive function, while others report limited or no associations (Table 6).

Nauta et al. observed that reductions in psychological symptoms mediated improvements in self-reported cognitive complaints and executive functioning but did not extend to objective cognitive measures such as information processing speed [35]. Similarly, Lincoln et al. demonstrated that CR improved psychological symptoms (as measured by GHQ-30) at both 6- and 12-month follow-ups, with positive correlation with reduced memory problems in daily life. Psychological symptoms improvements were associated with reduced subjective cognitive complaints, suggesting an indirect link between cognitive interventions and psychological well-being [30,31]. Hanssen et al. reported significant improvements in psychological well-being within the intervention group after four months, with trends toward continued improvement at seven months, indicating a potential connection between CR and reduced psychological distress [33]. Leonardi et al. found that both VRRS and traditional CR improved psychological symptoms (measured by BDI), with VRRS-associated cognitive gains linked to enhanced psychological symptoms and reduced anxiety levels [44]. Similarly, Impellizzeri et al. highlighted that enhanced psychological symptoms and emotional awareness were related to better cognitive outcomes in the experimental group [49]. Grasso et al. reported that improvements in cognitive function were associated with reductions in depression scores, which persisted at six-month follow-up for the intervention group [40]. Stuifbergen et al. also found that participants in the intervention group reported reduced depressive symptoms (measured by CES-D) post-intervention compared to controls, emphasizing the interplay between improved cognition and reduced depression [36]. Maggio et al., Brenk et al., and Goverover et al. similarly reported that enhanced cognitive outcomes were associated with reductions in depressive symptoms, with Goverover et al. specifically noting significant depression reduction in the treatment group [27,28,45]. Rosti-Otajärvi et al. observed that interventions led to reduced depressive symptoms and fatigue levels, particularly among those with more severe attentional deficits, further supporting the relationship between CR and psychological well-being improvements [37].

However, not all studies found robust links; Jongen et al. reported significant improvements in anxiety and depression one month post-cognitive training but noted these effects were not sustained at later follow-ups [34]. Solari et al. found minimal changes in psychological symptoms scores with no significant relationship between cognitive and psychological outcomes; Hildebrandt et al. similarly reported no significant association between these domains [38,39].

Overall, while many studies suggest a positive relationship between CR and psychological outcomes in MS, variability exists across findings, highlighting the need for further research to clarify these interactions and their long-term implications. While both mindfulness-based and VR-based interventions improve psychological symptoms in pwMS, mindfulness may offer the most robust and sustained benefits for depression, anxiety, and psychological QoL [50,51]. VR-based CR is particularly effective for anxiety and engagement but may be less potent for depression. Both depression and anxiety are well-established, independent predictors of reduced QoL in pwMS [52]. Their presence can exacerbate the impact of CI on daily functioning and well-being. For instance, depression seems to directly lower QoL while being associated with worse cognitive outcomes, increased fatigue, and decreased self-efficacy. Anxiety may have an even greater impact on psychological symptoms and QoL than depression alone, especially when both are present [53].

## 9. Implications for Rehabilitation of People with Multiple Sclerosis

The findings of this review highlight several important implications for rehabilitation in pwMS. Personalized, individualized CR emerges as particularly promising approach to address the heterogeneous cognitive profiles and varying needs of pwMS. Evidence suggests that tailored interventions yield superior outcomes compared to standardized protocols, demonstrating effectiveness for improving QoL measures in pwMS [54,55,56,57,58,59,60,61]. To achieve better results, a team-based approach appears crucial to addressing the complex needs of pwMS. A multidisciplinary rehabilitation team, comprising physiatrists, neurologists and various rehabilitation professionals (e.g., psychologists, neuropsychologists, physiotherapists, occupational therapists, speech therapists, and psychomotor therapists) play a critical role in the care of pwMS, with the final aim to achieve realistic functional goals [62,63,64]. Individual rehabilitation projects (IRPs) are critical in this context; they allow for the tailoring of goals, interventions, and intensity based on each patient’s unique neurological deficits, disease stage, and personal circumstances, thereby maximizing the potential for functional gains and accommodating variable response profiles observed in clinical trials [65,66,67]. The development of IRP for pwMS with CI requires a structured, evidence-based approach that integrates personalized assessments, goal-oriented interventions, and multidisciplinary coordination. Emerging evidence underscores the importance of tailoring CR to address the heterogeneous cognitive profiles and psychosocial needs of this population, while leveraging validated tools and therapeutic modalities to optimize functional outcomes [5,68]. The foundation of any IRP lies in a detailed comprehensive evaluation to characterize the type and severity of disabilities. In case of CI, this assessment should comprise a neuropsychological evaluation. Studies emphasize the utility of domain-specific assessments, such as the Luria 10-word memorization technique and Schulte tables, which have demonstrated diagnostic value in identifying mild cognitive dysfunction and tracking disease progression [69]. Recent advances in cognitive phenotyping, as outlined in the International Classification of Cognitive Disorders in MS, provide a taxonomy that categorizes patients into distinct subgroups based on impairment patterns-ranging from isolated processing speed deficits to multi-domain involvement [6]. Longitudinal data suggest these phenotypes remain relatively stable over one year, supporting their use as a basis for personalized intervention plans. For example, patients classified under “phenotype 3” (marked visuo-executive and memory deficits) may benefit from targeted executive function training, while those in “phenotype 2” (mild attention impairments) might prioritize sustained attention exercises [68,70]. Effective rehabilitation frameworks incorporate patient-centered goal-setting methodologies such as goal attainment scaling, which allows for the quantification of progress toward individualized objectives [71,72].

Traditional and GCR approaches are well-established, with evidence supporting improvements in memory, attention, and executive functions. Group-based interventions may offer added psychosocial benefits through peer support, but effects on QoL are variable and often modest. CCR and digital interventions appears feasible, scalable, and show consistent cognitive benefits, especially for processing speed and attention. VR-based CR is emerging as a highly engaging approach with evidence of superior cognitive and QoL outcomes compared to traditional methods in some studies. VR may enhance motivation, adherence, and transfer of skills to daily life, but requires specialized equipment and training. Combining CR with modalities like music therapy or mindfulness may yield additive benefits for both cognition and psychological well-being, though evidence is still preliminary. Most robust evidence for combined cognitive, QoL, and psychological benefits is seen with individualized, multidisciplinary approaches that integrate compensatory strategies, technology (computer-based or VR), and psychosocial support. Feasibility varies by setting: CCR and GCR are widely implementable, while VR and integrative approaches are promising but may be limited by resource availability. The choice of modality should be tailored to patient needs, available resources, and clinical context.

Several possible interventions could thus be included in an IRP for pwMS in the case of CI. Among these, individualized compensatory programs, such as the self-generation learning protocol, have demonstrated efficacy in improving memory encoding and functional status by teaching patients to actively reconstruct information rather than passively receive it [28,73]. CCR platforms offer scalable, home-based solutions for MS-related cognitive deficits. Meta-analyses indicate that CCR significantly improves processing speed and attention, particularly when using adaptive programs like RehaCom or Lumosity, which adjust task difficulty based on real-time performance [74,75]. Hybrid models combining in-person sessions with tele-rehabilitation may optimize adherence and long-term outcomes, as seen in studies where brief telephone follow-ups reinforced goal achievement [76,77]. These findings underscore the importance of integrating cognitive phenotyping, personalized goal setting, and hybrid digital approaches into routine rehabilitation care for people with MS. A personalized, multidisciplinary approach should be not only a theoretical recommendation but a compassionate, patient-centered strategy for addressing the complex cognitive and emotional challenges experienced by pw MS.

Many interventions showed short-term improvements but the evidence for sustained, long-term benefits is mixed and often limited by short follow-up periods and methodological variability. Several studies included in this review report that cognitive and psychosocial gains tend to diminish over time if not actively maintained. For example, Lincoln et al. found that improvements in memory and mood observed at six months were not sustained at 12 months. Similarly, Jongen et al. reported transient QoL improvements one month post intervention, with no significant differences at six months. A recent meta-analysis highlighted the general lack of studies with long-term follow-up (beyond one year), underscoring a critical gap in the literature and the need for research into strategies that promote durable outcomes [75]. Promoting the maintenance of gains may require booster sessions, the integration of strategies into daily life, personalized and multidisciplinary care, and leveraging technology for ongoing support [78,79].

## 10. Conclusions

In conclusion, CR appears promising in addressing cognitive impairments associated with MS, with notable implications for enhancing QoL. This review highlights the multifaceted benefits of CR approaches, spanning traditional, group-based, computer-assisted, and innovative methods, on various QoL domains. While the effectiveness of CR in improving cognitive performance is well-documented, its impact on QoL outcomes reveals a more nuanced picture, influenced by intervention type, duration, and individual variability.

Several studies demonstrate that cognitive improvements achieved through CR are often accompanied by enhancements in health-related QoL, particularly in mental health and functional domains. Notable interventions such as VR-based training and music therapy have shown a positive influence in both cognitive and psychosocial outcomes (e.g., anxiety and depression), underscoring the potential of integrative approaches to address the complex needs of pwMS. However, other studies report limited or transient QoL benefits, suggesting that sustained improvements may require personalized strategies that address not only cognitive deficits but also emotional and social dimensions. However, even if VR and music therapy appears as promising modalities, the evidence is preliminary and derived from small cohorts. The interpretation of their efficacy should be cautious, particularly in light of limited long-term follow-up.

The psychological outcomes of CR warrant special attention. Many interventions have shown a positive influence on anxiety and depression, which are critical components of overall well-being in MS populations. Mindfulness-based therapies and self-generation learning programs have demonstrated reductions in depressive symptoms and anxiety while fostering emotional resilience.

As limitations, it should be pointed out that QoL in pwMS is inherently a subjective and multidimensional concept, shaped by a constellation of factors that can vary significantly between individuals (e.g., perceived cognitive efficacy, mood and psychological well-being, functional and social changes). Each study tended to prioritize or measure QoL differently, often reflecting the specific aims of the intervention, the instruments used, and the domains most relevant to their participants. As a result, synthesizing a unified, operational definition of QoL across all trials was not possible, undermining the overall evaluation of CR effects in this setting. Moreover, many papers showed short intervention periods, lack of blinded outcome assessment, and inconsistent cognitive phenotyping.

Future research should focus on tailoring interventions to individual needs, exploring long-term effects on QoL, and identifying mechanisms that link cognitive improvements to psychological well-being. The promise of CR in MS is compelling, but rigorous trials with standardized outcomes and extended follow-up are necessary to establish its long-term benefits across cognitive, emotional, and functional domains. By addressing these gaps, CR can be further optimized to improve not only cognitive functioning but also the broader aspects of QoL.

## Figures and Tables

**Table 1:** Summary of traditional CR features.

Key Point	Detail
**Focus of Traditional CR**	Emphasizes compensatory strategies (internal/external aids, repetition, structured exercises) for adaptation, not restoration.
**Individual vs. Group CR**	Individual CR: tailored, flexible, close therapist–patient relationship; group CR: social stimulation, mutual support.
**Evidence from Studies**	Mixed results.
**Cognitive Domain Improvements**	Some interventions improve short-term/working memory, attention, visuo-constructive skills, and reduce depression.
**QoL Effects**	Inconsistent: some studies show improvements, others no significant change.
**Psychological Effects**	Some interventions reduce depressive symptoms and improve mood, especially in the short term; effects are variable and may not be sustained in the long-term.

**Table 2:** Summary of group CR features.

Key Point	Detail
**Focus of Group CR**	Enhances cognitive functioning in structured group settings; emphasizes social interaction, mutual support, and shared coping strategies to reduce isolation and foster empowerment.
**Group CR vs.** **Individual CR**	Group CR: promotes communication, relational skills, and social stimulation; individual CR: more tailored and flexible. Choice depends on patient needs, goals, and resources.
**Evidence from Studies**	Mixed results across studies.
**Cognitive Domain Improvements**	Some interventions improve memory (short-term, working, verbal), attention, executive functions, and self-reported cognitive functioning. Gains are often domain-specific and may not generalize to all cognitive areas.
**QoL Effects**	Inconsistent: some studies show improvements (especially with added follow-up or emotional support), others report no significant change.
**Psychological** **Effects**	Certain interventions reduce depression, anxiety, and improve psychological well-being and self-efficacy, but effects are variable and sometimes not statistically significant.

**Table 3:** Summary of CCR features.

Key Point	Detail
**Focus of CCR**	Utilizes digital platforms and computer-based exercises to target cognitive deficits (memory, attention, executive functions) in pwMS. Technology accessibility and familiarity facilitate integration into re-educational programs.
**CCR vs. Traditional/Group CR**	CCR offers structured, standardized, and often home-based or self-paced interventions; can be personalized and widely accessible. It may lack the social interaction of group CR but provides flexibility and scalability.
**Evidence from Studies**	Generally positive, with most studies reporting improvements in targeted cognitive domains and some reporting benefits in mood and QoL. However, results can vary by program, intensity, and outcome measured.
**Cognitive Domain Improvements**	Multiple studies show gains in memory, attention, working memory, executive control, and processing speed. Effects are often domain-specific; improvements in some areas (e.g., memory, attention) may not generalize to all cognitive functions.
**QoL Effects**	Mixed: Some studies report significant improvements in QoL (e.g., MAPSS_MS, RehaCom, *Dr. Kawashima’s Brain Training*), while others find no significant change despite cognitive gains.
**Psychological Effects**	Several interventions reduce depressive symptoms, particularly in those with more severe baseline deficits. Some studies report improved self-efficacy and daily functioning, but broader psychosocial impact is inconsistent.

**Table 4:** Summary of VR features.

Key Point	Detail
**Focus of Virtual Reality (VR) CR**	Employs immersive 2D/3D environments to simulate real-world settings for cognitive and motor re-education in pwMS. Enhances patient engagement, motivation, and provides enriched sensory feedback to stimulate neuroplasticity.
**VR CR vs. Traditional/Computer CR**	VR offers a more engaging, interactive, and realistic experience than traditional CR or CCR. Allows real-time feedback and dynamic task adjustment but may require specialized equipment and training.
**Evidence from Studies**	Generally positive: studies report significant improvements in global cognitive functioning, learning, memory, executive functions, visuospatial skills, and motor function, often exceeding traditional CR.
**Cognitive Domain Improvements**	VR interventions improve attention, memory (short-term, verbal), executive functions, visuospatial skills, information processing speed, and learning ability. Effects are often broader and more pronounced than with traditional CR.
**QoL Effects**	Several studies report significant improvements in QoL, particularly in mental health-related domains, following VR-based interventions. Gains are often more marked than with traditional CR.
**Psychological Effects**	VR-based CR can reduce depression and improve mood and self-efficacy. Enhanced motivation and reduced isolation are also reported, especially when VR is combined with tele-rehabilitation or multisensory feedback.

**Table 5:** Summary of cognitive and QoL outcomes relation in pwMS.

Key Point	Detail
**General Relationship**	The link between CR and QoL in pwMS is complex, with studies showing mixed and sometimes inconsistent results.
**Positive Associations**	Several studies report that improvements in cognitive domains (e.g., memory, learning, executive function) are associated with better mental health-related QoL, general health, and self-reported cognitive abilities.
**Short-Term vs. Long-Term Effects**	Some interventions yield short-term QoL improvements (up to 6 months), but these effects are often not sustained at longer follow-ups (12 months or more).
**Variability by Intervention Type**	The impact of CR on QoL varies by intervention type (traditional, group, computer-based, VR), with some approaches (e.g., VR, multidisciplinary, follow-up support) showing stronger or broader effects.
**Influence of Psychological Factors**	Improvements in psychological well-being, mood, and reduced psychological symptoms often mediate QoL gains, sometimes more than direct cognitive changes.
**Lack of Consistent Correlation**	Several studies found no significant relationship between cognitive gains and QoL improvements, or only weak/indirect associations, indicating that factors other than cognition may play a key role in QoL outcomes.
**Individual Variability**	Outcomes are influenced by individual factors such as baseline impairment, intervention duration, and personal goals; personalized approaches may yield more sustained and meaningful benefits.
**Personalization**	Long-term and robust improvements in both cognition and QoL often require interventions tailored to address both cognitive deficits and psychosocial needs of pwMS.

**Table 6:** Summary of cognitive and psychological outcomes relation in PwMS.

Key Point	Summary/Detail
**General Relationship**	The relationship between CR and psychological outcomes in pwMS is multifaceted, with studies reporting both direct and indirect effects, as well as some inconsistent findings.
**Positive Associations**	Many studies report that CR leads to significant improvements in psychological well-being, including reductions in depression, anxiety, and psychological distress, often mediated by improvements in cognitive function.
**Indirect Effects**	Improvements in psychological symptoms are frequently linked to reduced subjective cognitive complaints, indicating that psychological benefits may arise indirectly through perceived cognitive gains.
**Sustained vs. Short-Term Effects**	Some interventions yield sustained reductions in depression and psychological symptoms (up to six months), while others observe only short-term benefits that diminish over time.
**Variability by Intervention Type**	Both traditional and technology-based CR (e.g., VR) can improve psychological outcomes, with some evidence suggesting greater effects when cognitive gains are pronounced, or interventions are multidisciplinary.
**Influence of Baseline Characteristics**	Greater psychological improvements are often observed in individuals with more severe baseline cognitive or attentional deficits, suggesting that patient characteristics influence outcomes.
**Lack of Consistent Correlation**	Several studies found minimal or no significant association between cognitive gains and psychological improvements, indicating that not all CR interventions yield robust psychological benefits.

## Data Availability

No new data were created or analyzed in this study. Data sharing is not applicable to this article.

## Supplementary Materials

The following supporting information can be downloaded at: https://www.mdpi.com/article/10.3390/neurosci6030064/s1. Table S1: Data from analyzed papers.

## References

[1] Manocchio N., Argento O., Bossa M., Spanò B., Pellicciari L., Foti C., Nocentini U. (2025). The Role of the Cerebellum in Multiple Sclerosis-Related Fatigue and Disability. J. Clin. Med..

[2] Beer S., Khan F., Kesselring J. (2012). Rehabilitation Interventions in Multiple Sclerosis: An Overview. J. Neurol..

[3] Tramontano M., Argento O., Manocchio N., Piacentini C., Orejel Bustos A.S., De Angelis S., Bossa M., Nocentini U. (2024). Dynamic Cognitive–Motor Training versus Cognitive Computer-Based Training in People with Multiple Sclerosis: A Preliminary Randomized Controlled Trial with 2-Month Follow-Up. J. Clin. Med..

[4] Benedict R.H.B., Amato M.P., DeLuca J., Geurts J.J.G. (2020). Cognitive Impairment in Multiple Sclerosis: Clinical Management, MRI, and Therapeutic Avenues. Lancet Neurol..

[5] Bossa M., Manocchio N., Argento O. (2022). Non-Pharmacological Treatments of Cognitive Impairment in Multiple Sclerosis: A Review. NeuroSci.

[6] Hancock L.M., Galioto R., Samsonov A., Busch R.M., Hermann B., Matias-Guiu J.A. (2023). A Proposed New Taxonomy of Cognitive Phenotypes in Multiple Sclerosis: The International Classification of Cognitive Disorders in MS (IC-CoDiMS). Mult. Scler. J..

[7] Ramaglia V., Dubey M., Malpede M.A., Petersen N., De Vries S.I., Lee D.S.W., Schenk G.J., Gold S.M., Huitinga I., Gommerman J.L. (2021). Complement-Associated Loss of CA2 Inhibitory Synapses in the Demyelinated Hippocampus Impairs Memory. Acta Neuropathol..

[8] Nabizadeh F., Balabandian M., Rostami M.R., Owji M., Sahraian M.A., Bidadian M., Ghadiri F., Rezaeimanesh N., Moghadasi A.N. (2022). Association of Cognitive Impairment and Quality of Life in Patients with Multiple Sclerosis: A Cross-Sectional Study. Curr. J. Neurol..

[9] Genov K., Dimitrova M. (2022). Correlation between Cognitive Abilities and Social Functioning in Patients with Multiple Sclerosis. Folia Medica.

[10] Marafioti G., Cardile D., Culicetto L., Quartarone A., Lo Buono V. (2024). The Impact of Social Cognition Deficits on Quality of Life in Multiple Sclerosis: A Scoping Review. Brain Sci..

[11] Henry A., Lannoy S., Chaunu M., Tourbah A., Montreuil M. (2022). Social Cognition and Executive Functioning in Multiple Sclerosis: A Cluster-analytic Approach. J. Neuropsychol..

[12] Zameer U., Tariq A., Asif F., Kamran A. (2024). Empowering Minds and Bodies: The Impact of Exercise on Multiple Sclerosis and Cognitive Health. Ann. Neurosci..

[13] Feinstein A. (2006). Mood Disorders in Multiple Sclerosis and the Effects on Cognition. J. Neurol. Sci..

[14] Lin X., Zhang X., Liu Q., Zhao P., Zhong J., Pan P., Wang G., Yi Z. (2021). Empathy and Theory of Mind in Multiple Sclerosis: A Meta-Analysis. Front. Psychiatry.

[15] Khaligh-Razavi S.-M., Sadeghi M., Khanbagi M., Kalafatis C., Nabavi S.M. (2020). A Self-Administered, Artificial Intelligence (AI) Platform for Cognitive Assessment in Multiple Sclerosis (MS). BMC Neurol..

[16] Longley W.A. (2022). Cognitive Rehabilitation in Multiple Sclerosis. Aust. J. Gen. Pract..

[17] Bossa M., Argento O., Piacentini C., Manocchio N., Scipioni L., Oddi S., Maccarrone M., Nocentini U. (2023). Effects of Long-Term Oral Administration of N-Palmitoylethanolamine in Subjects with Mild Cognitive Impairment: Study Protocol. Brain Sci..

[18] Novakovic-Agopians T., Abrams G.M. (2014). Cognitive Rehabilitation Therapy. Encyclopedia of the Neurological Sciences.

[19] De Meo E., Portaccio E., Bonacchi R., Giovannoli J., Niccolai C., Amato M.P. (2025). An Update on the Treatment and Management of Cognitive Dysfunction in Patients with Multiple Sclerosis. Expert Rev. Neurother..

[20] Serruya M.D., Kahana M.J. (2008). Techniques and Devices to Restore Cognition. Behav. Brain Res..

[21] De Luca R., Calabrò R.S., Bramanti P. (2018). Cognitive Rehabilitation after Severe Acquired Brain Injury: Current Evidence and Future Directions. Neuropsychol. Rehabil..

[22] Dong Q., Yang Y., Tang Q., Yang M., Lan A., Xiao H., Wei J., Cao X., Xian Y., Yang Q. (2023). Effects of Early Cognitive Rehabilitation Training on Cognitive Function and Quality of Life in Critically Ill Patients with Cognitive Impairment: A Randomised Controlled Trial. Aust. Crit. Care.

[23] Locke D.E.C., Cerhan J.H., Wu W., Malec J.F., Clark M.M., Rummans T.A., Brown P.D. (2008). Cognitive Rehabilitation and Problem-Solving to Improve Quality of Life of Patients with Primary Brain Tumors: A Pilot Study. J. Support. Oncol..

[24] Medalia A., Saperstein A.M., Hansen M.C., Lee S. (2018). Personalised Treatment for Cognitive Dysfunction in Individuals with Schizophrenia Spectrum Disorders. Neuropsychol. Rehabil..

[25] Clare L., Kudlicka A., Oyebode J.R., Jones R.W., Bayer A., Leroi I., Kopelman M., James I.A., Culverwell A., Pool J. (2019). Individual Goal-oriented Cognitive Rehabilitation to Improve Everyday Functioning for People with Early-stage Dementia: A Multicentre Randomised Controlled Trial (the GREAT Trial). Int. J. Geriatr. Psychiatry.

[26] Lincoln N.B. (2002). Evaluation of Cognitive Assessment and Cognitive Intervention for People with Multiple Sclerosis. J. Neurol. Neurosurg. Psychiatry.

[27] Brenk A., Laun K., Haase C.G. (2008). Short-Term Cognitive Training Improves Mental Efficiency and Mood in Patients with Multiple Sclerosis. Eur. Neurol..

[28] Goverover Y., Chiaravalloti N., Genova H., DeLuca J. (2018). A Randomized Controlled Trial to Treat Impaired Learning and Memory in Multiple Sclerosis: The Self-GEN Trial. Mult. Scler. J..

[29] Mani A., Chohedri E., Ravanfar P., Mowla A., Nikseresht A. (2018). Efficacy of Group Cognitive Rehabilitation Therapy in Multiple Sclerosis. Acta Neurol. Scand..

[30] Lincoln N.B., Bradshaw L.E., Constantinescu C.S., Day F., Drummond A.E., Fitzsimmons D., Harris S., Montgomery A.A., Das Nair R. (2020). Group Cognitive Rehabilitation to Reduce the Psychological Impact of Multiple Sclerosis on Quality of Life: The CRAMMS RCT. Health Technol. Assess..

[31] Lincoln N.B., Bradshaw L.E., Constantinescu C.S., Day F., Drummond A.E., Fitzsimmons D., Harris S., Montgomery A.A., das Nair R., Morgan M. (2020). Cognitive Rehabilitation for Attention and Memory in People with Multiple Sclerosis: A Randomized Controlled Trial (CRAMMS). Clin. Rehabil..

[32] Brissart H., Omorou A.Y., Forthoffer N., Berger E., Moreau T., De Seze J., Morele E., Debouverie M. (2020). Memory Improvement in Multiple Sclerosis after an Extensive Cognitive Rehabilitation Program in Groups with a Multicenter Double-Blind Randomized Trial. Clin. Rehabil..

[33] Hanssen K.T., Beiske A.G., Landrø N.I., Hofoss D., Hessen E. (2016). Cognitive Rehabilitation in Multiple Sclerosis: A Randomized Controlled Trial. Acta Neurol. Scand..

[34] Jongen P.J., Van Mastrigt G.A., Heerings M., Visser L.H., Ruimschotel R.P., Hussaarts A., Duyverman L., Valkenburg-Vissers J., Cornelissen J., Bos M. (2019). Effect of an Intensive 3-Day Social Cognitive Treatment (Can Do Treatment) on Control Self-Efficacy in Patients with Relapsing Remitting Multiple Sclerosis and Low Disability: A Single-Centre Randomized Controlled Trial. PLoS ONE.

[35] Nauta I.M., Van Dam M., Bertens D., Kessels R.P.C., Fasotti L., Uitdehaag B.M.J., Speckens A.E.M., De Jong B.A. (2024). Improved Quality of Life and Psychological Symptoms Following Mindfulness and Cognitive Rehabilitation in Multiple Sclerosis and Their Mediating Role for Cognition: A Randomized Controlled Trial. J. Neurol..

[36] Stuifbergen A.K., Becker H., Perez F., Morrison J., Brown A., Kullberg V., Zhang W. (2018). Computer-Assisted Cognitive Rehabilitation in Persons with Multiple Sclerosis: Results of a Multi-Site Randomized Controlled Trial with Six Month Follow-Up. Disabil. Health J..

[37] Rosti-Otajärvi E., Mäntynen A., Koivisto K., Huhtala H., Hämäläinen P. (2013). Neuropsychological Rehabilitation Has Beneficial Effects on Perceived Cognitive Deficits in Multiple Sclerosis during Nine-Month Follow-Up. J. Neurol. Sci..

[38] Solari A., Motta A., Mendozzi L., Pucci E., Forni M., Mancardi G., Pozzilli C. (2004). Computer-Aided Retraining of Memory and Attention in People with Multiple Sclerosis: A Randomized, Double-Blind Controlled Trial. J. Neurol. Sci..

[39] Hildebrandt H., Lanz M., Hahn H.K., Hoffmann E., Schwarze B., Schwendemann G., Kraus J.A. (2007). Cognitive Training in MS: Effects and Relation to Brain Atrophy. Restor. Neurol. Neurosci..

[40] Grasso M.G., Broccoli M., Casillo P., Catani S., Pace L., Pompa A., Rizzi F., Troisi E. (2017). Evaluation of the Impact of Cognitive Training on Quality of Life in Patients with Multiple Sclerosis. Eur. Neurol..

[41] De Giglio L., De Luca F., Prosperini L., Borriello G., Bianchi V., Pantano P., Pozzilli C. (2015). A Low-Cost Cognitive Rehabilitation with a Commercial Video Game Improves Sustained Attention and Executive Functions in Multiple Sclerosis: A Pilot Study. Neurorehabil Neural Repair..

[42] Maggio M.G., De Luca R., Molonia F., Porcari B., Destro M., Casella C., Salvati R., Bramanti P., Calabro R.S. (2019). Cognitive Rehabilitation in Patients with Traumatic Brain Injury: A Narrative Review on the Emerging Use of Virtual Reality. J. Clin. Neurosci..

[43] Russo M., Dattola V., De Cola M.C., Logiudice A.L., Porcari B., Cannavò A., Sciarrone F., De Luca R., Molonia F., Sessa E. (2018). The Role of Robotic Gait Training Coupled with Virtual Reality in Boosting the Rehabilitative Outcomes in Patients with Multiple Sclerosis. Int. J. Rehabil. Res..

[44] Leonardi S., Maggio M.G., Russo M., Bramanti A., Arcadi F.A., Naro A., Calabrò R.S., De Luca R. (2021). Cognitive Recovery in People with Relapsing/Remitting Multiple Sclerosis: A Randomized Clinical Trial on Virtual Reality-Based Neurorehabilitation. Clin. Neurol. Neurosurg..

[45] Maggio M.G., De Luca R., Manuli A., Buda A., Foti Cuzzola M., Leonardi S., D’Aleo G., Bramanti P., Russo M., Calabrò R.S. (2022). Do Patients with Multiple Sclerosis Benefit from Semi-Immersive Virtual Reality? A Randomized Clinical Trial on Cognitive and Motor Outcomes. Appl. Neuropsychol. Adult.

[46] Chatterjee D., Hegde S., Thaut M. (2021). Neural Plasticity: The Substratum of Music-Based Interventions in Neurorehabilitation. NeuroRehabilitation.

[47] Phillips C.S., Kim J., Ganesh A., Stuifbergen A.M. (2025). Impact of Active Versus Receptive Music Interventions on Psychosocial and Neurological Outcomes in People with Multiple Sclerosis: A Systematic Review. J. Integr. Complement. Med..

[48] T Zaatar M., Alhakim K., Enayeh M., Tamer R. (2024). The Transformative Power of Music: Insights into Neuroplasticity, Health, and Disease. Brain Behav. Immun. Health.

[49] Impellizzeri F., Leonardi S., Latella D., Maggio M.G., Foti Cuzzola M., Russo M., Sessa E., Bramanti P., De Luca R., Calabrò R.S. (2020). An Integrative Cognitive Rehabilitation Using Neurologic Music Therapy in Multiple Sclerosis: A Pilot Study. Medicine.

[50] Culicetto L., Lo Buono V., Donato S., La Tona A., Cusumano A.M.S., Corello G.M., Sessa E., Rifici C., D’Aleo G., Quartarone A. (2024). Importance of Coping Strategies on Quality of Life in People with Multiple Sclerosis: A Systematic Review. J. Clin. Med..

[51] Komar A., Dickson K., Alavinia M., Bruno T., Bayley M., Feinstein A., Scandiffio J., Simpson R. (2024). Effects of Mindfulness-Based Interventions on Cognition in People with Multiple Sclerosis: A Systematic Review and Meta-Analysis of Randomized Controlled Trials. Front. Psychiatry.

[52] Margoni M., Preziosa P., Rocca M.A., Filippi M. (2023). Depressive Symptoms, Anxiety and Cognitive Impairment: Emerging Evidence in Multiple Sclerosis. Transl. Psychiatry.

[53] Hanna M., Strober L.B. (2020). Anxiety and Depression in Multiple Sclerosis (MS): Antecedents, Consequences, and Differential Impact on Well-Being and Quality of Life. Mult. Scler. Relat. Disord..

[54] Chiaravalloti N.D., Genova H.M., DeLuca J. (2015). Cognitive Rehabilitation in Multiple Sclerosis: The Role of Plasticity. Front. Neurol..

[55] Munger K.C., Martinez A.P., Hyland M.H. (2021). The Impact of Cognitive Rehabilitation on Quality of Life in Multiple Sclerosis: A Pilot Study. Mult. Scler. J. Exp. Transl. Clin..

[56] Jeong I.C., Karpatkin H., Finkelstein J., Honey M., Ronquillo C., Lee T.-T., Westbrooke L. (2021). Physical Telerehabilitation Improves Quality of Life in Patients with Multiple Sclerosis. Studies in Health Technology and Informatics.

[57] Tacchino A., Podda J., Bergamaschi V., Pedullà L., Brichetto G. (2023). Cognitive Rehabilitation in Multiple Sclerosis: Three Digital Ingredients to Address Current and Future Priorities. Front. Hum. Neurosci..

[58] Agostini F., Pezzi L., Paoloni M., Insabella R., Attanasi C., Bernetti A., Saggini R., Mangone M., Paolucci T. (2021). Motor Imagery: A Resource in the Fatigue Rehabilitation for Return-to-Work in Multiple Sclerosis Patients—A Mini Systematic Review. Front. Neurol..

[59] Etoom M., Khraiwesh Y., Lena F., Hawamdeh M., Hawamdeh Z., Centonze D., Foti C. (2018). Effectiveness of Physiotherapy Interventions on Spasticity in People with Multiple Sclerosis: A Systematic Review and Meta-Analysis. Am. J. Phys. Med. Rehabil..

[60] Calabrò R.S., Cassio A., Mazzoli D., Andrenelli E., Bizzarini E., Campanini I., Carmignano S.M., Cerulli S., Chisari C., Colombo V. (2021). What Does Evidence Tell Us about the Use of Gait Robotic Devices in Patients with Multiple Sclerosis? A Comprehensive Systematic Review on Functional Outcomes and Clinical Recommendations. Eur. J. Phys. Rehabil. Med..

[61] Restivo D.A., Quartarone A., Bruschetta A., Alito A., Milardi D., Marchese-Ragona R., Iezzi E., Peter S., Centonze D., Stampanoni Bassi M. (2024). Dysphagia in Multiple Sclerosis: Pathophysiology, Assessment, and Management—An Overview. Front. Neurol..

[62] Khalfaoui S. (2024). The Role of Physical Medicine and Rehabilitation in the Management of Multiple Sclerosis: A Review of the Literature. J. Phys. Med. Rehab. Stud. Rep..

[63] Magro V.M., Sorbino A., Manocchio N., Ljoka C., Foti C. (2025). The Physiatrist in Intensive Care: Role, Tasks, and Critical Issues in a Clinical Case Report Analysis. Clin. Transl. Neurosci..

[64] Manocchio N., Magro V.M., Massaro L., Sorbino A., Ljoka C., Foti C. (2025). Hashimoto’s Encephalopathy: Clinical Features, Therapeutic Strategies, and Rehabilitation Approaches. Biomedicines.

[65] Manocchio N., Ljoka C., Ferdinandi V., Cicchi L., Foti C. (2025). Commentary on “The Learning Rehabilitation System: Strengthening an Intersectoral Strategy to Improve Functioning of an Ageing Population” by Bickenbach et al. Health Policy.

[66] Argento O., Piacentini C., Bossa M., Caltagirone C., Santamato A., Saraceni V., Nocentini U. (2023). Motor, Cognitive, and Combined Rehabilitation Approaches on MS Patients’ Cognitive Impairment. Neurol. Sci..

[67] Homayuni A., Abedini S., Hosseini Z., Etemadifar M., Ghanbarnejad A. (2021). Explaining the Facilitators of Quality of Life in Patients with Multiple Sclerosis: A Qualitative Study. BMC Neurol..

[68] Podda J., Di Antonio F., Tacchino A., Pedullà L., Grange E., Battaglia M.A., Brichetto G., Ponzio M. (2024). A Taxonomy of Cognitive Phenotypes in Multiple Sclerosis: A 1-Year Longitudinal Study. Sci. Rep..

[69] Andreichenko D.I., Kalbus O.I. (2024). Diagnostic Value of the Luria 10-Word Memorization Technique and Schulte Tables in the Diagnosis of Mild Cognitive Dysfunction in Patients with Multiple Sclerosis. VPBM.

[70] De Meo E., Portaccio E., Giorgio A., Ruano L., Goretti B., Niccolai C., Patti F., Chisari C.G., Gallo P., Grossi P. (2021). Identifying the Distinct Cognitive Phenotypes in Multiple Sclerosis. JAMA Neurol..

[71] Rannisto M., Rosti-Otajärvi E., Mäntynen A., Koivisto K., Huhtala H., Hämäläinen P. (2015). The Use of Goal Attainment Scaling in Neuropsychological Rehabilitation in Multiple Sclerosis. Disabil. Rehabil..

[72] Pradeau C., Estival S., Postal V., Laurier V., Maugard C., Isner-Horobeti M.-E., Mourre F., Krasny-Pacini A. (2025). A Pilot Rating System to Evaluate the Quality of Goal Attainment Scales Used as Outcome Measures in Rehabilitation. Neuropsychol. Rehabil..

[73] Goverover Y., Chiaravalloti N., DeLuca J. (2008). Self-Generation to Improve Learning and Memory of Functional Activities in Persons with Multiple Sclerosis: Meal Preparation and Managing Finances. Arch. Phys. Med. Rehabil..

[74] Naser Moghadasi A., Mirmosayyeb O., Bagherieh S., Sahraian M.A., Ghajarzadeh M. (2025). Computerized Cognitive Rehabilitation in Patients with Multiple Sclerosis (MS): A Systematic Review and Meta-Analysis. Casp. J. Intern. Med..

[75] Rayegani S.M., Heidari S., Seyed-Nezhad M., Kiyani N., Moradi-Joo M. (2024). Effectiveness of Cognitive Rehabilitation in Comparison with Routine Rehabilitation Methods in Patients with Multiple Sclerosis: A Systematic Review and Meta-Analysis. Mult. Scler. J. Exp. Transl. Clin..

[76] Pagliari C., Di Tella S., Jonsdottir J., Mendozzi L., Rovaris M., De Icco R., Milanesi T., Federico S., Agostini M., Goffredo M. (2024). Effects of Home-Based Virtual Reality Telerehabilitation System in People with Multiple Sclerosis: A Randomized Controlled Trial. J. Telemed. Telecare.

[77] Schröder J., Van Criekinge T., Embrechts E., Celis X., Van Schuppen J., Truijen S., Saeys W. (2019). Combining the Benefits of Tele-Rehabilitation and Virtual Reality-Based Balance Training: A Systematic Review on Feasibility and Effectiveness. Disabil. Rehabil. Assist. Technol..

[78] Barbarulo A.M., Lus G., Signoriello E., Trojano L., Grossi D., Esposito M., Costabile T., Lanzillo R., Saccà F., Morra V.B. (2018). Integrated Cognitive and Neuromotor Rehabilitation in Multiple Sclerosis: A Pragmatic Study. Front. Behav. Neurosci..

[79] De Gier M., Beckerman H., Twisk J.W., Knoop H., De Groot V. (2024). Effectiveness of a Blended Booster Programme for the Long-Term Outcome of Cognitive Behavioural Therapy for MS-Related Fatigue: A Randomized Controlled Trial. Mult. Scler. J..

