# Supplementary material for: Beyond Cognition: Cognitive Re-Education’s Impact on Quality of Life and Psychological Well-Being in People with Multiple Sclerosis—A Narrative Review
Source: NeuroSci. 2025 Jul 15;6(3):64. doi: 10.3390/neurosci6030064 (PMC12286144; doi:10.3390/neurosci6030064)
Supplement: Supplementary file 1 [file neurosci-06-00064-s001.zip › neurosci-3693677-supplementary.pdf]

Literature Search Strategy

A search of the literature was conducted using the following search strategy on the PubMed database: "(non-pharmacological treatment cognitive impairment multiple sclerosis) OR (therapies of cognitive impairment in multiple sclerosis) OR (treatment of cognitive impairment multiple sclerosis) OR (treatment of cognitive disorders in multiple sclerosis) OR (rehabilitation of cognitive impairment multiple sclerosis) OR (rehabilitation of cognitive disorders multiple sclerosis) AND (quality of life)". No other filters were applied, and no time limit was considered, to broaden the search.

Table S1: Data from analyzed papers

Authors, year	Title	Study Type	Sample Size	Sex	Age: mean, (range)	Type of disease	Re-educational Treatment	Outcome Measure (Cog)	Outcome Measure (QoL)	Results	Cog and QoL outcomes relation	Outcome measure (PsY)	Cog ad PsY outcomes relation

Brenk, 2008 [27]	Short-term cognitive training improves mental efficiency and mood in patients with multiple sclerosis.	Case Control Study	27 MS patients, 14 healthy controls	F= 53,65% M = 46,35%	MS group = 43.5 (25-59) Control subject = 39.6 (24 - 55)	RRMS	Home-based 6-week cognitive training program targeting memory, attention, planning, and verbal performance (selected exercises from 'Gripsgymnastik/ Brain-Gym' as non-specific cognitive training that targets memory and attention functions, as well as planning and verbal performances)	Verbal repeating of numbers forward (ZNS-V) and backwards (ZNS-R). Wechsler Memory Scale, by the verbal learn-ing and memory test (VLMT) Rey-Osterrieth complex figure test (CFT). Test battery of attention (TAP). To assess executive functions, the Regensburger test of word flency (RWT). Additional executive functions and visuo-constructive performances were investigated by the Rey-Oster-rieth CFT.	HAQUAMS	Significant improvement in short-term and working memory, visuoconstructive abilities, and figural long-term memory; depression and QoL improved during training and persisted up to 6 months. Verbal long-term memory showed some deterioration.	+	BDI (Beck's depression inventory)	+

Brissart H, 2020 [32]	Memory improvement in multiplesclerosis after an extensivescleros is after an extensive cognitive rehabilitation program in groups with a multicenter double-blind randomized trial	RCT	128	F = 77.2% M = 22.8%	46,1	RRMS e PSM	ProCog-SEP: 13 group sessions over 6 months, including psychoeducational advice and cognitive exercises. Placebo: non-cognitive exercises with no strategies or advice.	Selective Reminding Test (verbal memory), Digit Span Backward, Working Memory omissions, others.	Multiple Sclerosis International Quality of Life Questionnaire	ProCog-SEP was more effective in increasing in learning index and in working memory on manipulation, and updating capacities. No difference was observed for other neuropsychological outcomes. Regarding quality of life outcomes, no change was observed between the two groups	-	/	/

De Giglio. L, 2014 [41]	A Low-Cost Cognitive Rehabilitation With a Commercial Video Game Improves Sustained Attention and Executive Functionsin Multiple Sclerosis: A Pilot Study	RCT	35 (18 interventio n, 17 control)	F = 74% M = 26%	43, 9 (18-50)	RRMS	Home-based cognitive rehabilitation using Dr. Kawashima's Brain Training (DKBT), 8 weeks, 30 min/day, 5 days/week	ST (Stroop Test) PASAT (Paced Auditory Serial Addition Test) SDMT (Symbol Digit Modalities Test).	MSQoL-54	Significant improvements in ST and SDMT; trends for PASAT and cognitive fatigue improvement; QoL improvements in mental health composite score, role limitations-emotional, emotional well-being, cognitive function, and health distress subscales. No significant effects on other QoL subscales.	+	/	/

Goverover Y, 2018 [28]	A randomized controlled trial to treat impaired learning and memory in multiple sclerosis: The self-GEN trial.	RCT	35	F = 25,7 % M= 74,3 %	49 (31-65)	RRMS = 69 % SPMS= 11 % PPMS = 20%	Self-generation learning program (self-GEN trial)	CMT (Contextual Memory Test) MIST (Memory for Intentions Test) MFQ (Memory Functioning Questionnaire) SRSI (the Self-Regulation Skills Interview) CVLT-II (California Verbal Learning Test–2nd Edition)	FBP (Functional behavioral profile) FAMS Functional Assessment of Multiple Sclerosis (FAMS),	Significant improvements in memory performance (CMT, MIST), self-regulation (SRSI), and quality of life (FAMS). Depression decreased in the treatment group. No significant changes noted on CVLT-II or general self-awareness.	+	STAI: State-Trait Anxiety Inventory; CMDI: Chicago Multiscale Depression Inventory	+
Grasso MG, 2017 [40]	Evaluation of the Impact of Cognitive Training on Quality of Life in Patients with Multiple Sclerosis.	RCT	34	F = 64% M = 36%	59 (47-65)	RRMS = 47,1% SPMS = 44,1% PPMS = 8,8 %	Cognitive training (APT-based) + multidisciplinary rehabilitation vs. multidisciplinary rehabilitation alone	Stroop Color Word Interference Test (SCWIT), Rao's Brief Repeatable Battery tests (Selective Reminding Test, 10/36 Spatial Recall Test, Symbol Digit Modalities Test, Paced Auditory Serial Addition	SF-36	Multidisciplinary intervention group improved in executive function (SCWIT, p=0.024) and QoL domains including GH, VT, BP, SF, MC, and PC. MD group improved only in physical composite	+	Montgomery and Asberg Depression Rating Scale (MADRS), Fatigue Severity Scale (FSS)	+

								Test, Word List Generation)		score. Depression improved in both groups but persisted longer in CMD group.			
Hanssen KT, 2016 [33]	Cognitive rehabilitation in multiple sclerosis: a randomized controlled trial	RCT	120	F = 80% in control group, 66.7% in interventi on group	53.2 (32-71)	RRMS (53.3% control, 45% interventi on) SPMS (30% control, 25% interventi on) PPMS (16.7% control, 30% interventi on)	Multicompone nt cognitive rehabilitation program (4-week inpatient multidisciplina ry rehabilitation + cognitive group/individu al sessions + Goal Attainment Scaling [GAS] + biweekly telephone follow-up for 3 months)	BRIEF - A (Behavior Rating Inventory of Executive Function for adults)	HRQoL (Health-related quality of life)	Both groups had a slight improvement in BREAF-A of approximatly 1 SD above the normative mean. Intervention group show an improvement close to significantly more tha the control group, on QoL aspect. HSCL-25 improved but not significantly.	+	HSCL-25 (Hopkins Symptom Checklist-25)	+

Hildebrandt H. 2007 [39]	Cognitive training in MS: Effects and relation to brain atrophy	RCT	42	F= 57 % M= 43 %	39 (23 - 63)	RRMS	Home-based cognitive training program targeting memory and working memory, 6 weeks, 5 days/week, 30 mins/day. Patients received a compact disk (CD) with memory and work-ing memory rehabilitation tasks (VILAT-G 1.0)	CVLT (California Verbal Learning treatment) PASAT	SF-36	Improved verbal learning, long-delay verbal memory, and working memory in the treatment group. No significant effects on QoL or fatigue. Training effects on cognitive outcomes were partly influenced by brain atrophy levels.	-	BDI	-
Impellizzeri F, 2020 [49]	An integrative cognitive rehabilitation using neurologic music therapy in multiple sclerosis: A pilot study.	RCT	30	F = 37% M = 63%	51 Range: Not provided	RRMS = 50% PPSM = 25% SPMS = 25%	1- Control Group received conventional Cognitive Rehabilitation for 6 times a week for 8 weeks (n=15) 2- Intervention Group received Cognitive rehabilitation +	BRB-N (battery of neuropsychological test)	MSQoL-54	EG showed significant improvements in cognitive functions (e.g., long-term storage/retrieval and delayed recall) and mental QoL compared to CG. Improvements	+	Beck Depression Inventory (BDI), Emotion Awareness Questionnaire (EAQ).	+

							Neurological Musical terapy (NMT) 3 times a week for 8 weeks each (n=15)			in emotional status, motivation, and mood were also more pronounced in the EG.			
Jongen PJ, 2019 [34]	Effect of an intensive 3-day social cognitivereatment (can do treatment) on control self-efficacy in patients with relapsing remitting multiple sclerosis and low disability: A single- centre randomized controlled trial	RCT	158	F=87.9%, M=12.1%	40 (20-61)	RRMS	3-day intensive "Can Do Treatment" (CDT)	MSSES-C (Multiple Sclerosis Self-Efficacy Scale Control) self-efficacy MSSES-F IPA (Impact on Participation and Autonomy questionnaire)	MSQoL-54	No significant improvement in MSSES-C at 6 months; significant improvement at 1 and 3 months in CDT group; control group improved gradually over time.	-	HADS (Hospital Anxiety and Depression Scale)	-

Leonardi S, 2021 [44]	Cognitive recovery in people with relapsing/remitting multiple sclerosis A randomized clinical trial on virtual reality-based neurorehabilitation.	RCT	30 (15 Conventional Cognitive Rehabilitation and 15 Virtual reality rehabilitation)	F = 40% M= 60%	54.6 (52 - 56)	RRMS	Virtual Reality Rehabilitation System (VRRS) vs. Conventional Cognitive Rehabilitation (CR)	MOCA (Montreal Cognitive Assessment), BRB-N (Global Cognition) SRT_LTS (Long Term Storage - Verbal Memory) SRT-CLTR (Consistent Long term - Retrieval Verbal Memory) SPART (Visual-spatial Memory) SDMT (Attention Processes)	MSQoL-54	Both VRRS and CR improved mood and visuospatial skills. Only VRRS significantly enhanced global cognition, learning ability, short-term verbal memory, lexical access ability, and mental health-related QoL.	+	BDI (Beck Depression Inventory) HRS-A (Hamilton Rating scale Anxiety)	+
Lincoln NB, 2020 [30]	Group cognitive rehabilitation to reduce the psychological impact of multiple sclerosis on quality of life: the CRAMMS RCT.	RCT	449	F = 73% M = 27%	49 (18-69)	RRMS (65%) PPMS (10%) PSMS (25%)	Group cognitive rehabilitation program with 10 weekly sessions focusing on restitution and compensatory strategies	EMQ (Everyday Memory questionnaire) BRBN (Brief Repeatable Battery of Neuropsychological Tests)	MusiQoL (Multiple Sclerosis International Quality of Life questionnaire)	The results indicate that there was no benefit of this cognitive rehabilitation program for this group of people with MS on their quality of life. There was a difference	-	General Health Questionnaire-30 (GHQ-30)	+

Lincoln NB, 2020 [31]	Cognitive rehabilitation for attention and memory in people with multiple sclerosis: a randomized controlled trial (CRAMMS).	RCT	449	F= 73% M= 27%	49 (18-69)	RRMS (65%) PPMS (10%) PSMS (25%)	Group cognitive rehabilitation program with 10 weekly sessions focusing on restitution and compensation strategies for memory and attention. Usual care was the control.	EMQ (Everyday Memory questionnaire) BRBN (Brief Repeatable Battery of Neuropsychological Tests)	Multiple Sclerosis Impact Scale Psychological Subscale, EQ-5D-5L	between the cognitive rehabilitation and the usual-care groups in terms of subjective participants and related reports of memory problems, as assessed on the EMQ, favoring the cognitive rehabilitation group at both 6 and 12 months Cognitive rehabilitation showed no long-term effects on QoL at 12 months but had short-term benefits at 6 months. Improved mood and reduced subjective memory complaints	-	General Health Questionnaire-30 (GHQ-30)	+

										were observed at both 6 and 12 months.			
Lincoln NB, 2002 [26]	Evaluation of cognitive assessment and cognitive intervention for people with multiple sclerosis.	RCT	240	F = 65% M= 35%	43	RRMS 48% SPMS 38% PPMS 14%	Cognitive assessment and intervention (e.g., diaries, mnemonics), feedback to patients, carers, and professionals	DEX (subjective reports of dysexecutive syndrome) EMQ (everyday memory problems) MAQ (frequency of memory aids used)	GHQ-28 SF-36 Overall QoL (OQoL), Satisfaction with QoL (SQoL)	No significant effect of cognitive assessment or intervention on mood, QoL, independence, or subjective cognitive impairment. Minor differences favored the control group.	-	/	/

Maggio MG, 2022 [45]	Do patients with multiple sclerosis benefit from semi-immersive virtual reality? A randomized clinical trial on cognitive and motor outcomes.	RCT	60	F = 48,3 % M = 51,7%	50 (39 - 61)	RRMS SPMS	Semi-immersive Virtual Reality Training (sVRT) using BTS Nirvana system for experimental group; traditional cognitive rehabilitation for control group. Both groups had 24 sessions over 8 weeks.	MoCA (Montreal Cognitive Assessment) SPART e ROCF-DL (memory) PASAT (attention)	MSQoL-54	Significant improvement in cognitive parameters, particularly in visuospatial memory, executive functions, attention, and information processing speed for the experimental group (sVRT). Quality of life scores improved significantly in the experimental group compared to controls.	+	BDI (Depression)	+

Nauta IM, 2024 [35]	Improved quality of life and psychological symptoms following mindfulness and cognitive rehabilitation in multiple sclerosis and their mediating role for cognition: a randomized controlled trial	RCT	99	M = 26% F= 73%	48,8 (18-65)	RRMS 64% SPMS 18% PPMS 5% Unclear 5%	CRT =Cognitive Rehabilitation Therapy (n = 32 pazienti) MBCT = Mindfulness based cognitive therapy (n = 32 pazienti) ETAU = Controlled group (n= 35 pazienti)	CFQ (Cognitive Failure Questionnaire) BRIEF-A (Behavior Rating Inventory of Executive Function-Adult Version) IPS (Individual Placement and Support) GAS (Goal Attainment Scaling)	MSQoL-54	MBCT and CRT improved mental QoL and reduced depressive symptoms; MBCT also reduced fatigue and brooding. Effects on self-reported cognition were mediated by psychological symptoms and mindfulness skills but not for objective cognition. Effects diminished at 6 months except for mindfulness skills after MBCT.	+	HADS (Hospital Anxiety and Depression Scale) CIS-20 (Checklist Individual Strength) RRS-NL (Dutch Ruminative Response Scale) Mindfulness	+

Rosti-Otajärvi E, 2013 [37]	Neuropsychological rehabilitation has beneficial effects on perceived cognitive deficits in multiple sclerosis during nine-month follow-up	RCT	102	F = 80% M= 20%	44 (18 - 59)	RRMS	Strategy-oriented neuropsychological rehabilitation: weekly 60-minute sessions for 13 weeks, focusing on attention and working memory strategies, psychoeducation, and psychological support.	PDQ (Perceived Deficits Questionnaire) MSQLI (Multiple Sclerosis Quality of Life Inventory) MSNQ (ltiple Sclerosis Neuropsychological Questionnaire)	WHOQOL-BREF	Significant reduction in perceived cognitive deficits in the intervention group compared to controls at six months and one year. Improvements were more pronounced in patients with moderate to severe attentional deficits. No significant improvements in QoL measures at group level but individual goal achievements noted.	-	Beck Depression Inventory II (BDI-II), Multiple Sclerosis Impact Scale - Psychological Subscale (MSIS-29), Fatigue Scale for Motor and Cognitive Fatigue (FSMC)	+

Solari A. 2004 [38]	Computer-aided retraining of memory and attention in people with multiple sclerosis: a randomized, double-blind controlled trial	RCT	77	F = 63 % M = 37%	44 (22-65)	RRMS = 51% PMS = 49%	Computer-aided retraining of memory and attention vs. visuo-constructional and visuo-motor coordination training (control group)	BRBNT (It provides measures of sustained attention and concentration (paced auditory serial addition task [PASAT], and symbol digit modalities test), verbal learning and delayed recall (selective reminding), visuospatial learning and delayed recall (10/36 spatial recall), and phonemic retrieval (word list generation).	MSQoL-54	No significant difference in primary cognitive outcomes. Improvement seen in Word List Generation test for study group only. No significant improvement in other cognitive tests compared to the control group.	+	CMDI (Chicago mood depression inventory)	-

Stuifbergen AK, 2018 [36]	Computer-assisted cognitive rehabilitation in persons with multiple sclerosis: Results of a multi-site randomized controlled trial with six month follow-up	RCT	183	F = 87% M = 13%	49,6 (20-60)	RRMS = 69% PPSM = 8% SPMS = 23%	MAPSS - MS (Memory, Attention, Problem Solving Skills in MS) = the intervention includes group sessions (2 h per week for 8 weeks) focused on building efficacy for use of cognitive strategies and a home-based computer training program (45 min three times per week	MACFIMS (Minimal Assessment of Cognitive Function in MS) COWAT (Controlled Oral Word Association Test) CVLT-II (California Verbal Learning Test) BVMT-R (the Brief Visuospatial Memory Test – Revised) SDMT (The Paced Auditory Serial Addition Test)	Everyday Problems Test-Revised (EPT-R)	Intervention group showed significant improvement in delayed verbal memory (CVLT-II), processing speed (PASAT), and cognitive abilities (PROMIS) compared to controls. Improvements persisted at 3 and 6 months post-intervention. Control group also improved but to a lesser extent.	+	CESD	+
